# Carrier mobilities and electron-phonon interactions beyond DFT

**DOI:** 10.1038/s41524-026-02011-2

**Published:** 2026-03-03

**Authors:** Aleksandr Poliukhin, Nicola Colonna, Francesco Libbi, Samuel Poncé, Nicola Marzari

**Affiliations:** 1 Theory and Simulation of Materials (THEOS), École polytechnique fédérale de Lausanne, Lausanne, Switzerland; 2 PSI Center for Scientific Computing, Theory and Data, Villigen PSI, Switzerland; 3 John A. Paulson School of Engineering and Applied Sciences, Harvard University, Cambridge, MA USA; 4 European Theoretical Spectroscopy Facility, Institute of Condensed Matter and Nanosciences, Université catholique de Louvain, Louvain-la-Neuve, Belgium; 5 WEL Research Institute, Wavre, Belgium

**Keywords:** Materials science, Physics

## Abstract

Electron-phonon coupling is a key interaction that governs diverse physical processes such as carrier transport, superconductivity, and optical absorption. Calculating such interactions from first-principles with methods beyond density-functional theory remains a challenge. We introduce here a finite-difference framework for computing electron-phonon couplings for any electronic structure method that provides eigenvalues and eigenvectors, and showcase applications for hybrid and Koopmans functionals, and *GW* many-body perturbation theory. Our approach introduces a novel projectability scheme based on eigenvalue differences and bypasses many of the limitations of the direct finite difference methods. It also leverages symmetries to reduce the number of independent atomic displacements, decreasing overall computational cost. This approach enables seamless integration with established first-principles codes for generating displaced supercells, performing Wannier interpolations, and evaluating transport properties. Applications to silicon and gallium arsenide show that advanced electronic-structure functionals predict different electron-phonon couplings and modify band curvatures, resulting in much more accurate estimates of intrinsic carrier drift mobilities and effective masses. In general, our method provides a robust and accessible framework for calculating the electron-phonon properties with state-of-the-art beyond DFT methods.

## Introduction

Electron-phonon interactions play a central role in determining fundamental materials’ properties, such as electron and hole mobilities^1–3^, superconductivity^4^, band renormalization^5^, and non-adiabatic effects^6^. Accurate modeling of these interactions is essential for advancing technologies ranging from efficient electronic devices to novel superconducting materials. First-principles calculations of electron-phonon couplings based on density-functional theory (DFT) have reached a maturity level that even allows high-throughput calculations^5^. A key step in these approaches is the evaluation of electron-phonon matrix elements, which quantify the effective interactions between electrons and phonons^4,7^. Computationally, two approaches exist for this task: density-functional perturbation theory (DFPT)^8,9^ and the finite difference (FD) method^10–12^. Recent work suggests machine learning as another promising alternative, as it allows accurate results when trained on relatively small datasets^13,14^. Although DFPT is generally a more computationally efficient approach than FD methods, as it offers favorable scaling and access to arbitrary phonon wavevectors **q**, it requires dedicated and involved implementations. For this reason, perturbative approaches based on DFPT have only recently been applied to specific beyond-DFT methods, such as DFT+U^15–17^ and *G*_0_*W*_0_^18,19^. Up to now, most of the calculations on the electron-phonon-related properties have been performed using DFPT with semilocal DFT functionals, and this method is considered to be the state of the art. Notwithstanding its success, discrepancies persist between first-principles predictions based on DFPT and experimental observations for many electron-phonon related properties^5,20,21^. This is partially due to the fact that Kohn-Sham (KS) DFT does not provide reliable quasiparticle energies and other excited-state properties^22–24^. To address this, more advanced methods have been developed to improve the description of quasiparticle energies. These include the incorporation of a fraction of exact exchange^25,26^, the enforcement of piecewise linearity conditions^27–32^, and the inclusion of many-body effects^18,33,34^. Improved electronic structure approaches were reported to affect electron-phonon properties^19,35,36^, but their impact on carrier mobility needs to be better understood. For these reasons, there is a growing need for a more straightforward and general method to calculate electron-phonon couplings. For a long time, finite differences have been successfully used to predict phonon properties with any functional, and today, well-established codes exist that implement these developments^37–39^. However, the absence of community codes to perform a similar task for electron-phonon properties underscores the need for a more accessible and general approach. One of the primary objectives of this work is to establish an approach based on FD to calculate electron-phonon matrix elements starting from any arbitrary electronic-structure method that provides eigenvectors and eigenvalues of the Hamiltonian for displaced systems. This approach and the code that implements it aim to fill the gap in the current software landscape. We illustrate our approach using Quantum ESPRESSO^40,41^, KOOPMANS^42^, and YAMBO^43^ as ab-initio electronic structure engines. Furthermore, we provide an interface to the EPW software^44,45^ and compute the transport properties of Si and GaAs to assess the effect that using hybrid and Koopmans functionals, and *GW* many-body perturbation theory have on transport properties.

## Results

The central quantity that describes electron-phonon interactions arises from the expansion of the effective potential that acts on the electrons due to the motion of atoms, which is given by:

1 $${\hat{H}}^{{\rm{e}}-{\rm{p}}{\rm{h}}}=\mathop{\sum }\limits_{\kappa \alpha l}\frac{\partial \hat{V}}{\partial {\tau }_{\kappa \alpha l}}\delta {\tau }_{\kappa \alpha l},$$

where $\hat{V}$ is the effective potential, *τ*_*καl*_ = **R**_*l*_ + *τ*_*κα*_ is the *α* Cartesian component of the position of the atom *κ* in a unit cell located at position **R**_*l*_. The matrix element of this operator describes the probability that an electron transitions from an initial to a final state because of the interaction with the atomic displacement *δτ*_*καl*_. Expanding the atomic displacement *δτ*_*καl*_ on the phonon basis, it is possible to define the electron-phonon matrix element *g*_*mnν*_(**k**, **q**) as:

2 $${g}_{mn\nu }({\bf{k}},{\bf{q}})\equiv \mathop{\sum }\limits_{\kappa \alpha l}\sqrt{\frac{\hslash }{2{M}_{\kappa }{\omega }_{{\bf{q}}\nu }}}{e}^{i{\bf{q}}\cdot {{\bf{R}}}_{l}}{e}_{\kappa \alpha {\bf{q}}\nu }\langle {\psi }_{m{\bf{k}}+{\bf{q}}}| \frac{\partial V}{\partial {\tau }_{\kappa \alpha l}}| {\psi }_{n{\bf{k}}}\rangle ,$$

where *ω*_**q***ν*_, *e*_*κα***q***ν*_ are the phonon frequencies and eigenvectors respectively, *ψ*_*n***k**_ is the one-particle wave function, and ∂*V*/∂*τ*_*καl*_ is the change of potential with respect to the displacement.

Standard FD approaches require one to evaluate the change in effective potential due to atomic displacement by using finite differences in a displaced supercell commensurate with the phonon **q**-vector of interest. However, in pseudopotential codes, there is a non-local part of the potential that is treated analytically and thus would require an additional implementation when different types of pseudopotentials are used, such as ultrasoft (US)^46^ or projector augmented wave (PAW)^47^ pseudopotentials. Moreover, in beyond-DFT approaches, such as hybrids, Koopmans, or *G*_0_*W*_0_, the effective potential is also a more complex operator that requires additional treatment, such as saving the unique potential for every orbital, which introduces additional memory constraints on the calculations. These challenges prevent the FD method from serving as a general-purpose approach for arbitrary electronic-structure functionals, as it would require distinct treatments for DFT and beyond-DFT methods, as well as for different pseudopotential schemes. To enable a unified approach to the calculation of electron-phonon couplings across different functionals, we propose here an alternative and general method that provides an easy and elegant solution to the challenge discussed above.

### Projectability approach based on eigenvalues

The derivative of the potential in Eq. (2) can be replaced by the derivative of the Hamiltonian, since the kinetic energy does not depend on the atomic position:

3 $$\begin{array}{rcl}\langle {\psi }_{m{\bf{k}}+{\bf{q}}}| \frac{\partial \hat{V}}{\partial {\tau }_{\kappa \alpha l}}| {\psi }_{n{\bf{k}}}\rangle & = & \langle {\psi }_{m{\bf{k}}+{\bf{q}}}| \frac{\partial \hat{H}}{\partial {\tau }_{\kappa \alpha l}}| {\psi }_{n{\bf{k}}}\rangle \\ & \approx & \langle {\psi }_{m{\bf{k}}+{\bf{q}}}| \frac{\hat{H}({\tau }_{\kappa \alpha l})}{2{\tau }_{\kappa \alpha l}}| {\psi }_{n{\bf{k}}}\rangle - \langle {\psi }_{m{\bf{k}}+{\bf{q}}}| \frac{\hat{H}(-{\tau }_{\kappa \alpha l})}{2{\tau }_{\kappa \alpha l}}| {\psi }_{n{\bf{k}}}\rangle .\end{array}$$

Using the first quantization representation of the Hamiltonian $\hat{H}={\sum }_{j}{\varepsilon }_{j}| {\psi }_{j}\rangle \langle {\psi }_{j}|$, one arrives at

4 $$\begin{array}{rcl}\langle {\psi }_{m{\bf{k}}+{\bf{q}}}| \frac{\partial V}{\partial {\tau }_{\kappa \alpha l}}| {\psi }_{n{\bf{k}}}\rangle & = & \frac{{\tau }_{\kappa \alpha l}^{-1}}{2}(\mathop{\sum }\limits_{j}{\varepsilon }_{j}^{+}{u}_{m{\bf{k}}+{\bf{q}}j}^{* +}{u}_{jn{\bf{k}}}^{+}\\ & & -\mathop{\sum }\limits_{j}{\varepsilon }_{j}^{-}{u}_{m{\bf{k}}+{\bf{q}}j}^{* -}{u}_{jn{\bf{k}}}^{-}),\end{array}$$

5 $${u}_{jn{\bf{k}}}^{+}=\langle {\psi }_{j}^{+}| {\psi }_{n{\bf{k}}}\rangle$$

6 $${u}_{jn{\bf{k}}}^{-}=\langle {\psi }_{j}^{-}| {\psi }_{n{\bf{k}}}\rangle ,$$

where ${\psi }_{j}^{\pm }$ and ${\varepsilon }_{j}^{\pm }$ are the wavefunction and eigenvalues of the perturbed supercell with band index *j* and **k** = **Γ**, and the + and − symbols represent positive and negative atomic displacement in Cartesian coordinates, respectively. Equation (4) represents the eigenvalue projectability approach for calculating electron-phonon matrix elements. Similar ideas were proposed for localized basis sets, such as LCAO^48^ or Wannier functions^49–51^, which have advantages in localizing perturbations in real space. However, constructing the perturbed Wannier functions in the supercells might not generally guarantee smoothness with respect to the unperturbed calculation. Moreover, the limiting factor in all the finite difference approaches is the self-consistent calculation of the ground state of the displaced supercell, meaning that any postprocessing step (as the one in Eq. (4)) represents just a fraction of the total computational time. Evaluation of brackets in Eq. (4) depends on the chosen basis set and the type of pseudopotential used. In the current work, we consider a plane-wave basis set with norm-conserving pseudopotentials for which the dot product is a sum over plane-wave components. For US and PAW pseudopotentials, the scalar product involves an overlap matrix that requires an additional treatment.

The difference between Eqs. (3) and (4) are that the latter allows for operating with the eigenvalues of the effective Hamiltonian instead of the Hamiltonian itself, which would be much more challenging to represent in a conventional real space approach (e.g., in the case of orbital-dependent potential of Koopmans and hybrid functionals or the non-local and frequency-dependent one of the GW method). Hence, the main advantage of Eq. (4) is that it only requires information about eigenvectors and eigenvalues of the pristine and displaced system, making the approach general and applicable to any beyond-DFT method as long as they deliver the quantities mentioned above. Established approaches^5^ allow for estimating electron-phonon coupling for specific points in the Brillouin zone using eigenvalue differences. Here, instead, by performing supercell calculations, one can automatically get information about electron-phonon matrix elements at any **q**-point commensurate with the size of the displaced supercell.

**Fig. 1:**
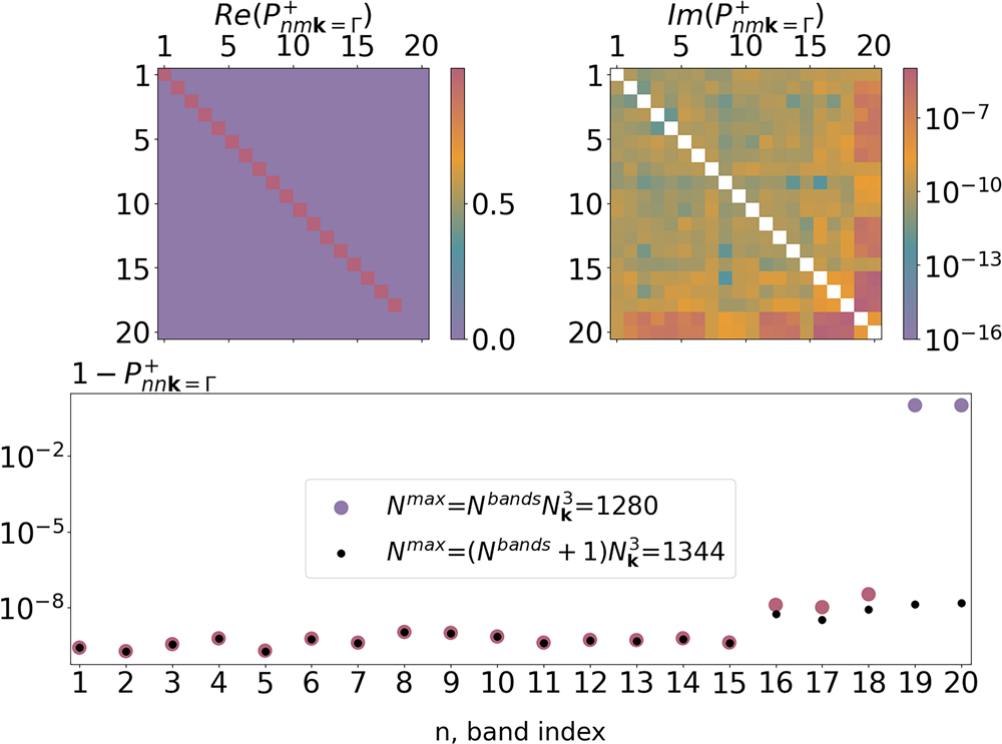
Verification of the projectability condition in Eqs. (7) and (8) for the 20 unperturbed states of silicon at *k* = *Γ.* The corresponding displaced supercell has dimensions 4 × 4 × 4, and ${N}^{\max }$ is set to 1280. The top left and top right are real and imaginary parts of the ${P}_{nm{\bf{k}}}^{+}$ matrix that should be close to identity. In this case, a good projectability holds only for the first 18 states. On the bottom panel, the diagonal part of the matrix is represented for the case when ${N}^{\max }$ = 1280 and ${N}^{\max }$ = 1344 are considered. Including all degenerate states (*n* = 19, 20, 21) allows for the fulfillment of the condition.

Two approximations are used in the derivation of Eq. (4): a finite difference formula for the evaluation of the change in the effective Hamiltonian, and the resolution of the identity that, in practical implementations, needs to be approximated with a finite set of eigenstates. The first approximation requires a convergence study with respect to the finite-difference step. Detailed information on the convergence of the FD approach can be found in SI^52^. To control the quality of the second approximation, we introduce the following unitary conditions:

7 $${P}_{nm{\bf{k}}}^{+}({N}^{\max })=\mathop{\sum }\limits_{j}^{{N}^{\max }}\langle {\psi }_{n{\bf{k}}}| {\psi }_{j}^{+}\rangle \langle {\psi }_{j}^{+}| {\psi }_{m{\bf{k}}}\rangle \approx {\delta }_{mn}$$

8 $${P}_{nn{\bf{k}}}^{+}({N}^{\max })=\mathop{\sum }\limits_{j}^{{N}^{\max }}| {u}_{jn{\bf{k}}}^{+}{| }^{2}\le 1,$$

where the sum runs over the eigenstates of the displaced system in the supercell setup up to a chosen threshold ${N}^{\max }$. Using perturbation theory for small displacements, it can be shown that if the unperturbed state *n***k** has a corresponding perturbed state *j* in Eq. (7), the error is ${\mathcal{O}}({\tau }^{2})$. Thus, if *N*^bands^ and *N*_**k**_ are the number of bands and the **k**-point sampling used for the pristine unit cell calculation repsectively, then one needs at least ${N}^{\max }={N}^{bands}{N}_{{\bf{k}}}^{3}$ states in the displaced supercell calculation with **Γ** sampling. However, in case the last unperturbed state is degenerate, more bands should be included in ${N}^{\max }$. The numerical evidence of this is presented in Fig. 1, where Eqs. (7) and (8) are illustrated for the 4 × 4 × 4 supercell of Si. In the first case, when we consider ${N}^{\max }$ = 1280, which corresponds to 20 unperturbed states, the projectability condition fails for the last 2 unperturbed states. This is because the unperturbed states 19, 20, and 21 are degenerate, and only two of the three states were included in the sum. Increasing ${N}^{\max }$ to 1344 (21 unperturbed states) brings the projectability condition in Eq. (7) back to a value close to 1. For an efficient evaluation of Eq. (4), one could also realize that most of the values in ${u}_{jm{\bf{k}}}^{\pm }$ are negligible since they represent the overlaps between perturbed and unperturbed wavefunctions with different quantum numbers. Note that the brackets in Eq. (5) are calculated between the unperturbed wave functions initially constructed in the unit cell and the wave functions of the supercell. For a consistent evaluation of the integral, unperturbed wave functions need to be unfolded in the supercell. After the unfolding, a Fourier transformation to reciprocal space can be performed, allowing for an efficient calculation of the brackets. We note that this unfolding procedure also allows us to keep track of the dependence of the electron-phonon matrix elements on **k** and **q** vectors in the primitive cell.

### Symmetries of displaced calculations

In order to evaluate electron-phonon matrix elements at finite **k** and **q** points, according to Eq. (2), we need to displace each atom in 3 Cartesian directions, performing in total 3*N*^at^*N*^uc^ supercell calculations, where *N*^at^ is the number of atoms in the unit cell, and *N*^uc^ is the number of unit cells in the supercell. Using the periodicity of the system, we have that Eq. (2) becomes^53^:

9 $${g}_{mn\nu }({\bf{k}},{\bf{q}})=N^{\mathrm{uc}}\mathop{\sum }\limits_{\kappa \alpha }\sqrt{\frac{\hslash }{2{M}_{\kappa }{\omega }_{{\bf{q}}\nu }}}{e}_{\kappa \alpha {\bf{q}}\nu }\langle {\psi }_{m{\bf{k}}+{\bf{q}}}| \frac{\partial V}{\partial {\tau }_{\kappa \alpha 0}}| {\psi }_{n{\bf{k}}}\rangle ,$$

where importantly ∂*V*/∂*τ*_*κα*_ needs to be evaluated only for a single reference unit cell in the supercell, reducing the number of displacements to 3*N*^at^. Evaluating the derivative with a second-order finite-difference scheme requires displacing the atoms in the + and − directions and increases the number of supercell calculations to 6*N*^at^. However, it is known^54^ that symmetry considerations for the case of phonon calculations allow reducing the number of displacements to *N*^in^, where *N*^in^ is the number of inequivalent atoms in the unit cell. For phonons, forces are calculated only for specific displacements and then rotated to obtain a complete set of displacements. In a perfect analogy, we consider a set of displacements {*λ*} that transform under the following conditions:

10 $$\hat{S}\lambda ={\lambda }^{{\prime} }$$

11 $$\hat{S}{R}_{\kappa }={R}_{{\kappa }^{{\prime} }},$$

where $\hat{S}$ represents the operator of space group symmetries that maps atom *κ* to atom ${\kappa }^{{\prime} }$. In this case, the potential evaluated at displacement ${\lambda }^{{\prime} }$ is related to that for displacement *λ* by a basis transformation:

12 $${V}^{{R}_{{\kappa }^{{\prime} }}+{\lambda }^{{\prime} }}({\bf{r}})={V}^{{R}_{\kappa }+\lambda }({\hat{S}}^{-1}{\bf{r}}).$$

Eq. (12) also implies that the Hamiltonian satisfies the same condition because the kinetic energy is invariant under unitary transformations of the basis. This means that the effective equations for the displacements of *λ* and ${\lambda }^{{\prime} }$ produce the same eigenvalues and are connected by:

13 $${\hat{H}}^{{R}_{{\kappa }^{{\prime} }}+{\lambda }^{{\prime} }}({\bf{r}}){\psi }_{n}^{{R}_{{\kappa }^{{\prime} }}+{\lambda }^{{\prime} }}({\bf{r}})={\varepsilon }_{n}{\psi }_{n}^{{R}_{{\kappa }^{{\prime} }}+{\lambda }^{{\prime} }}({\bf{r}})$$

14 $${\hat{H}}^{{R}_{\kappa }+\lambda }({\hat{S}}^{-1}{\bf{r}}){\psi }_{n}^{{R}_{\kappa }+\lambda }({\hat{S}}^{-1}{\bf{r}})={\varepsilon }_{n}{\psi }_{n}^{{R}_{\kappa }+\lambda }({\hat{S}}^{-1}{\bf{r}}).$$

Therefore, one performs only *N*^in^ displacements and then obtains the wavefunctions of the other displacements by symmetry ${\psi }_{n}^{{R}_{{\kappa }^{{\prime} }}+\hat{S}\lambda }({\bf{r}})={\psi }_{n}^{{R}_{\kappa }+\lambda }({\hat{S}}^{-1}{\bf{r}})$. An important caveat is that the displacements obtained by applying the symmetry operations have to be linearly independent when the same atom *κ* is considered. Indeed, Eq. (2) is written as the sum of derivatives of the potential along Cartesian displacements that would be connected to the set of displacement *λ*, *μ*, *ν* obtained by the symmetry operations:

15 $$\left[\begin{array}{rcl}{\lambda }_{x} & {\lambda }_{y} & {\lambda }_{z}\\ {\mu }_{x} & {\mu }_{y} & {\mu }_{z}\\ {\nu }_{x} & {\nu }_{y} & {\nu }_{z}\end{array}\right]\left[\begin{array}{l}\frac{\partial V}{\partial {x}_{\kappa }}\\ \frac{\partial V}{\partial {y}_{\kappa }}\\ \frac{\partial V}{\partial {z}_{\kappa }}\end{array}\right]=\left[\begin{array}{l}\frac{\partial V}{\partial {\lambda }_{\kappa }}\\ \frac{\partial V}{\partial {\mu }_{\kappa }}\\ \frac{\partial V}{\partial {\nu }_{\kappa }}\end{array}\right],$$

where the matrix on the left-hand side represents the transformation matrix between 3 independent displacements and 3 Cartesian displacements of atom *κ*. If any displacements are linearly dependent, the equation becomes undetermined.

### Electron-phonon coupling with realistic electrons

To show the simplicity and flexibility of our approach, we study the electron-phonon coupling of silicon at different levels of theory: DFT, HSE, Koopmans (KI), and *G*_0_*W*_0_. Since most observables require a dense **k** and **q** momentum integration of the electron-phonon matrix elements, we initially perform a calculation with a relatively small supercell and then use a Wannier interpolation approach^55–57^ to obtain smooth functions. Starting from the coarse grid calculation of electronic band structure, phonon dispersion, and electron-phonon coupling, the interpolation could be done by transforming back to reciprocal space the Wannier representation of the mentioned quantities:

16 $$\begin{array}{ll}{H}_{mn}({{\bf{R}}}_{p}) = & \frac{1}{{N}_{p}}\mathop{\sum }\limits_{{m}^{{\prime} }{n}^{{\prime} }{\bf{k}}}{e}^{-i{\bf{k}}\cdot {{\bf{R}}}_{p}}\\ & \times \, {U}_{m{m}^{{\prime} }{\bf{k}}}^{\dagger }{H}_{{m}^{{\prime} }{n}^{{\prime} }{\bf{k}}}{U}_{{n}^{{\prime} }n{\bf{k}}}\end{array}$$

17 $$\begin{array}{ll}{D}_{\kappa \alpha {\kappa }^{{\prime} }\beta } = & \frac{1}{{N}_{{p}^{{\prime} }}}\mathop{\sum }\limits_{\mu \nu {\bf{q}}}{e}^{-i{\bf{q}}\cdot {{\bf{R}}}_{{p}^{{\prime} }}}\\ & \times\, {e}_{\kappa \alpha {\bf{q}}\mu }^{\dagger }{D}_{\mu \nu {\bf{q}}}{e}_{{\kappa }^{{\prime} }\beta {\bf{q}}\nu }\end{array}$$

18 $$\begin{array}{ll}{g}_{mn\kappa \alpha }({{\bf{R}}}_{p},{{\bf{R}}}_{{p}^{{\prime} }}) = & \frac{1}{{N}_{p}{N}_{{p}^{{\prime} }}}\mathop{\sum }\limits_{{\bf{k}}{\bf{q}}}{e}^{-i({\bf{k}}\cdot {{\bf{R}}}_{p}+{\bf{q}}\cdot {{\bf{R}}}_{{p}^{{\prime} }})}\\ & \times\, \mathop{\sum }\limits_{{m}^{{\prime} }{n}^{{\prime} }\nu }\sqrt{\frac{2{M}_{\kappa }{\omega }_{{\bf{q}}\nu }}{\hslash }}{e}_{\kappa \alpha {\bf{q}}\nu }^{\dagger }\\ & \times {U}_{m{m}^{{\prime} }{\bf{k}}+{\bf{q}}}^{\dagger }{g}_{{m}^{{\prime} }{n}^{{\prime} }\nu }({\bf{k}},{\bf{q}}){U}_{{n}^{{\prime} }n{\bf{k}}},\end{array}$$

where *N*_*p*_ and ${N}_{p}^{{\prime} }$ are the numbers of the unit cells in the equivalent supercells for electron and phonons, ${D}_{\kappa \alpha {\kappa }^{{\prime} }\beta }$ is the dynamical matrix, ${U}_{{n}^{{\prime} }n{\bf{k}}}$ is a unitary rotation matrix that transforms the Bloch wave functions to a Wannier gauge. The maximum localization procedure can yield specific ${U}_{{n}^{{\prime} }n{\bf{k}}}$ matrices, which significantly improve interpolation quality by localizing Wannier functions in real space^55^. In order to efficiently use Wannier interpolation, the target quantity should be a smooth function of **k** and **q**, which is not the case for the electron-phonon coupling in polar materials because of the non-analytical behaviour at **q** = *Γ*. In this case, the electron-phonon matrix elements are decomposed into short- and long-range contributions:

19 $${g}_{mn\nu }({\bf{k}},{\bf{q}})={g}_{mn\nu }^{S}({\bf{k}},{\bf{q}})+{g}_{mn\nu }^{\mathcal{L}}({\bf{k}},{\bf{q}}).$$

The long range contribution ${g}_{mn\nu }^{{\mathcal{L}}}({\bf{k}},{\bf{q}})$ can be calculated from an analytical expression^58–60^:

20 $$\begin{array}{rcl}{g}_{mn\nu }^{{\mathcal{L}}}({\bf{k}},{\bf{q}}) & = & i\frac{{e}^{2}}{{V}^{\mathrm{uc}}{\varepsilon }^{0}}\mathop{\sum }\limits_{\kappa }\sqrt{\frac{\hslash }{2{N}_{{p}^{{\prime} }}{M}_{\kappa }{\omega }_{{\bf{q}}\nu }}}\\ & \times & \mathop{\sum }\limits_{{\bf{G}}\ne -{\bf{q}}}\frac{({\bf{q}}+{\bf{G}}){Z}_{\kappa }^{* }{{\bf{e}}}_{\kappa {\bf{q}}\nu }}{({\bf{q}}+{\bf{G}}){\varepsilon }^{\infty }({\bf{q}}+{\bf{G}})}\\ & \times & {e}^{-i({\bf{q}}+{\bf{G}})\cdot {\tau }_{\kappa }}\langle {\psi }_{m{\bf{k}}+{\bf{q}}}| {e}^{i({\bf{q}}+{\bf{G}})\cdot {\bf{r}}}| {\psi }_{n{\bf{k}}}\rangle ,\end{array}$$

where *V*^uc^ is the volume of the unit cell, *ε*^0^ is the vacuum dielectric constant, **ε**^*∞*^ is high-frequency dielectric tensor ${{\bf{Z}}}_{\kappa }^{* }$ is the Born effective charge tensor. It is important to state that long-range contribution is given in principle by infinite multipole expansion, and in this work, we only consider the dipole contribution^59,60^. Once the long-range contribution to the electron-phonon coupling is obtained, the conventional Wannier interpolation can be applied to the short-range component ${g}_{mn\nu }^{S}({\bf{k}},{\bf{q}})$, which is a smooth function in reciprocal space and therefore well suited for interpolation. As a final step, long-range contribution is added back to the interpolated short-range part, recovering the full expression for the electron-phonon coupling.

**Fig. 2:**
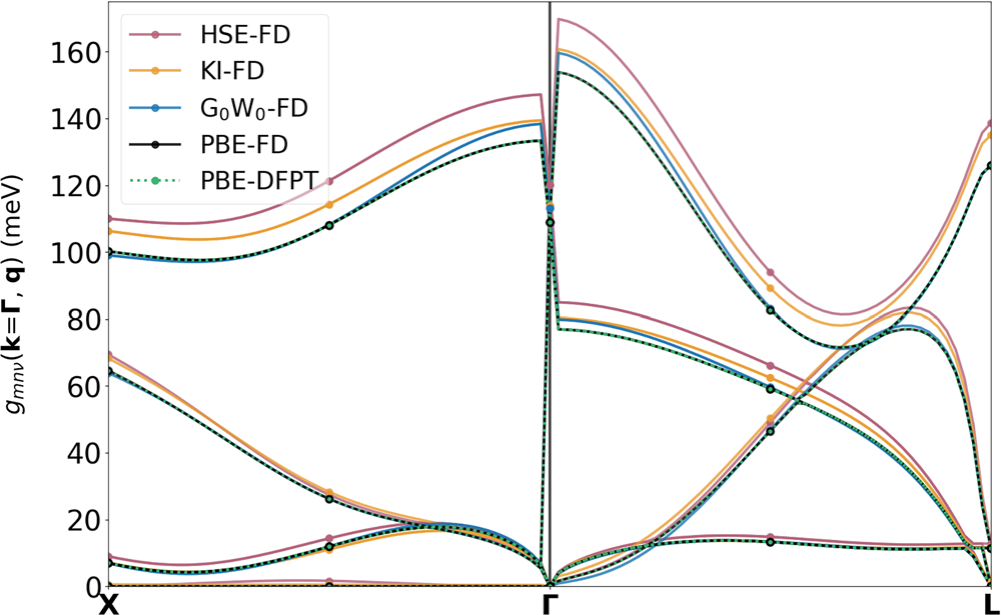
Interpolated electron-phonon matrix elements of Si along a q-momentum path at *k* = *Γ* for the electronic states of the second valence band (*m* = *n* = 2). All the results are interpolated with EPW using a coarse 4 × 4 × 4 **k/q** supercell. We show the result from a density-functional perturbation theory (DFPT) approach, interpolated from the same coarse grid, using a dashed green line. The direct results obtained using FD or DFPT on the 4 × 4 × 4 **k/q**-point grid are highlighted with circles. The PBE supercell finite difference (FD) and DFPT approach match perfectly, validating the approach.

The interpolated electron-phonon coupling of silicon for the band indices *m* = *n* = 2 along the high symmetry line ***X*** − **Γ** − ***L*** is shown in Fig. 2. The perfect agreement between DFPT and FD results for the standard semilocal PBE functional validates the projectability approach proposed here. Beyond DFT approaches show an increase in electron-phonon coupling compared to semilocal DFT, with HSE having an enhancement of 9%. This increase could be explained by the fact that DFT underestimates the electronic bandgap and overestimates the screening, leading to an overestimated dielectric constant. Since the electron-phonon matrix elements are inversely proportional to the screening^7^, such underestimation is expected. It is also important to stress that different electronic-structure methods produce different phonon spectra, as shown for the case of silicon in SI^52^ where the HSE functional is shown to increase the phonon frequencies on average by 5%. On the other hand, the KI functionals yield the same phonon dispersion as the DFT functional it is based on. This is because for insulating or semiconducting systems at integer electron number, the KI functional preserves the energetics (total energy and static derivatives of the total energy) of the underlying density-functional approximation. Therefore, KI forces are identical to DFT forces with the same interatomic force constant. Phonon properties with *G*_0_*W*_0_ are also kept at the DFT level, treating the eigenvalues correction in a perturbative way^19^. We note that in general DFT produces phonon dispersions in very good agreement with experiment^61^.

We find that the increase in electron-phonon matrix element is quite similar across phonon **q** and electron **k** (quasi-)momenta. The only exception in this case is *G*_0_*W*_0_ that tends to have a similar increase in coupling as in the Koopmans case close to *Γ*, but provides a negligible correction at finite **q**. Overall, the application of *G*_0_*W*_0_ corrections to the electron-phonon coupling still provides smooth corrections both as a function of **q** and **k** (see the SI^52^). The small values of the correction to the coupling obtained here are in good agreement with previous works on band renormalization in silicon^62^, although other calculations in the literature have reported more significant renormalization effects^63^. In the case of the band renormalization, the electron-phonon matrix elements enter as the sum over an infinite number of states, and converged values typically require hundreds of empty states^64^. That means that for band renormalizations, one should inspect the correction of the electron-phonon matrix elements in a broad range of indexes far from the Fermi level.

Given the smoothness of the corrections that beyond-DFT methods provide on top of DFT results, we introduce an effective scheme to accelerate the convergence for the calculation of the electron-phonon matrix elements with HSE, KI, and *G*_0_*W*_0_. We define a rescreening factor

21 $$R={\left|\left\langle \frac{{g}_{mn\nu }({\bf{k}},{\bf{q}})}{{g}_{mn\nu }^{\mathrm{DFT}}({\bf{k}},{\bf{q}})}\right\rangle \right|}^{2}$$

as the ratio between the beyond-DFT and the DFT electron-phonon matrix elements *g* averaged over all the bands and phonon indices, and **k** and **q** points. For the calculation of the ratio, the standard symmetrization procedure^56^ is applied: the absolute values of the couplings are averaged over degenerate manifolds, allowing the arbitrary phase to be neglected. The procedure is justified since most of the electron-phonon-related properties (e.g., transport, optical absorption, band gap renormalization) depend on the square modulus of the electron-phonon matrix elements. As shown in SI^52^, *R* converges fast with the **k** mesh, and can be evaluated from the electron-phonon matrix elements computed on relatively small supercells. We finally apply the rescreening factor to the *g*^DFT^ computed on a dense **k**/**q** mesh, getting very good estimates of the *g* for the beyond-DFT methods on the dense grid. Electronic states labeled by quasi-momentum **k** and band index *n* require only primitive cell calculations, and are evaluated directly on the same dense grid. This scheme allows us to avoid prohibitively expensive supercell calculations and, at the same time, obtain converged effective masses (key to getting well-converged transport properties). Even though we do not explicitly use electron-phonon matrix elements computed with a finite difference expression in the final calculation of mobility, all convergence tests with smaller supercells are performed using FD together with the Wannier interpolation of EPW.

### Phonon-limited transport

Phonon-limited transport is a key example in which electron-phonon interactions play a central role in determining the physical behavior of the system^3,65^. The electric current in semiconductors is related to the electric field **E** and the mobility tensor of electrons and holes *μ*_*αβ*_

22 $${\mu }_{\alpha \beta }=\mathop{\sum }\limits_{n}\int \frac{{d}^{3}{\bf{k}}}{{\Omega }^{\mathrm{BZ}}}{\upsilon }_{n{\bf{k}}\alpha }{\partial }_{{E}_{\beta }}{f}_{n{\bf{k}}}.$$

The latter can be computed using a linearized version of the Boltzmann transport equation (BTE)^66^ where the derivative of the distribution function *f*_*n***k**_ with respect to the external electric field is given by:

23 $$\begin{array}{ll}{\partial }_{{E}_{\beta }}{f}_{n{\bf{k}}} = & e{\upsilon }_{n{\bf{k}}\beta }\frac{\partial {f}_{n{\bf{k}}}^{0}}{\partial {\varepsilon }_{n{\bf{k}}}}{\tau }_{n{\bf{k}}}\\ & +\frac{2\pi {\tau }_{n{\bf{k}}}}{\hslash }\mathop{\sum }\limits_{m\nu }\int \frac{{d}^{3}{\bf{q}}}{{\Omega }^{\mathrm{BZ}}}| {g}_{mn\nu }({\bf{k}},{\bf{q}}){| }^{2}\\ & \times \left[ ({n}_{{\bf{q\nu }}}+1-{f}_{n{\bf{k}}}^{0})\right.\delta ({\varepsilon }_{n{\bf{k}}}-{\varepsilon }_{m{\bf{k}}+{\bf{q}}}+\hslash {\omega }_{{\bf{q}}\nu })\\ &\left. +({n}_{{\bf{q\nu }}}+{f}_{n{\bf{k}}}^{0})\delta ({\varepsilon }_{n{\bf{k}}}-{\varepsilon }_{m{\bf{k}}+{\bf{q}}}-\hslash {\omega }_{{\bf{q}}\nu })\right]{\partial }_{{E}_{\beta }}{f}_{m{\bf{k}}+{\bf{q}}},\end{array}$$

where *Ω*^BZ^ is the volume of the first BZ, ${\upsilon }_{n{\bf{k}}}={\hslash }^{-1}\frac{\partial {\varepsilon }_{n{\bf{k}}}}{\partial {\bf{k}}}$, ${f}_{n{\bf{k}}}^{0}$ is the Fermi-Dirac occupation function in equilibrium, and *n*_**qν**_ is Bose-Einstein distribution. The scattering rate ${\tau }_{n{\bf{k}}}^{-1}$ in Eq. (23) is

24 $$\begin{array}{ll}{\tau }_{n{\bf{k}}}^{-1} = & \frac{2\pi }{\hslash }\mathop{\sum }\limits_{m\nu }\int \frac{{d}^{3}{\bf{q}}}{{\Omega }^{\mathrm{BZ}}}| {g}_{mn\nu }({\bf{k}},{\bf{q}}){| }^{2}\\ & \times \left[({n}_{{\bf{q\nu }}}+1-{f}_{m{\bf{k}}+{\bf{q}}}^{0})\delta ({\varepsilon }_{n{\bf{k}}}-{\varepsilon }_{m{\bf{k}}+{\bf{q}}}-\hslash {\omega }_{{\bf{q}}\nu })\right.\\ & +\,\left.({n}_{{\bf{q\nu }}}+{f}_{m{\bf{k}}+{\bf{q}}}^{0})\delta ({\varepsilon }_{n{\bf{k}}}-{\varepsilon }_{m{\bf{k}}+{\bf{q}}}+\hslash {\omega }_{{\bf{q}}\nu })\right].\end{array}$$

It has been reported^67^ that the evaluation of mobility with the BTE requires very fine sampling near the edge of the band (typically of the order of 100^3^**k**- and **q**- grids), which is computationally prohibitive for both DFPT and FD approaches. A well established solution is to use Wannier-Fourier interpolations^55,56^, which allow to exploit the localization of the Wannier functions to perform an accurate interpolation starting from a coarse grid. Previous studies show^2^ that in the case where we do not consider additional scattering mechanisms in Eq. (23), the main factors that affect mobility are (i) the curvature of the electronic band structure (effective mass) near the band edges and (ii) the strength of the electron-phonon matrix elements. This means that a better description of the electronic band structure, for example, using beyond-DFT functionals, could improve the quality of the transport predictions.

We now investigate the effect of advanced electronic-structure approaches on the transport properties of Si and GaAs. For Si, we benchmark advanced functional approaches (KI and HSE) with state-of-the-art electronic structure simulation, namely G_0_W_0_, and find good agreement for both electron and hole effective masses across all the computational methods. For GaAs, we focused only on functional approaches, as converged *G*_0_*W*_0_ calculation would require hundreds of empty states in the primitive cell^68^, which is equivalent to tens of thousands in a 4 × 4 × 4 supercell. While this is still affordable for Si, the inclusion of *d*-semicore states in Ga and As pseudopotentials makes the calculation prohibitively expensive for GaAs.

As discussed previously, even though the drift mobility of Si is well captured when computed on DFT level^69–71^, the electron mobility of GaAs is significantly overestimated^71–73^, which could be improved by refining the effective mass description beyond DFT methods^70,74^ or by including higher order diagrams in the scattering term^75^. We report the electron and hole drift mobilities of silicon as well as electron drift mobilities of GaAs in Table 1 using the ab-initio iterative Boltzmann transport equation (BTE)^2,66^ and find that mobility with advanced electronic structure methods is reduced by up to 11% and 12% for electrons and holes, respectively, compared to DFT. The two main reasons behind this are the effect of rescreening of electron-phonon matrix elements and the change in the curvature of the electronic band structure. In the case of Si, these two effects have comparable contributions. As seen in Fig. 2, the re-screening is higher for the HSE hybrid functional compared to the case of Koopmans. However, the resulting mobility value is close to that of Koopmans, which has a smaller rescreening. The reason is that for the HSE functional, the change in the band structure curvature cancels the rescreening effect on the electron-phonon coupling, resulting in a carrier mobility comparable to that of the Koopmans functional. In the case of electron mobility with HSE and *G*_0_*W*_0_, both increase in the parallel component of the effective mass and electron-phonon coupling lower mobility. In all cases, we see that accounting for improved electronic description in the electron-phonon couplings improves the predictions of mobilities and brings them closer to the experimental values. See SI^52^ for the result on the temperature dependence of the mobility of Si beyond DFT methods.

**Table 1:** Electron and hole drift mobility of silicon and hole mobility of GaAs at 300 K using density-functional theory (DFT), hybrid functional (HSE), Koopman’s compliant functional (KI), and *G*_0_*W*_0_ using the iterative Boltzmann transport equation

	Drift mobility (cm^2^/Vs)				
	DFT	HSE	KI	*G*_0_*W*_0_	Experiment
${\mu }_{h}^{Si}$	517.0	486.1	483.7	461.2	445–501^94–96^
${\mu }_{e}^{Si}$	1552.7	1393.9	1412.3	1375.9	1350–1450^97,98^
${\mu }_{e}^{GaAs}$	50005.9	12800.8	15372.7	–	7200–9750^76–78^

**Fig. 3:**
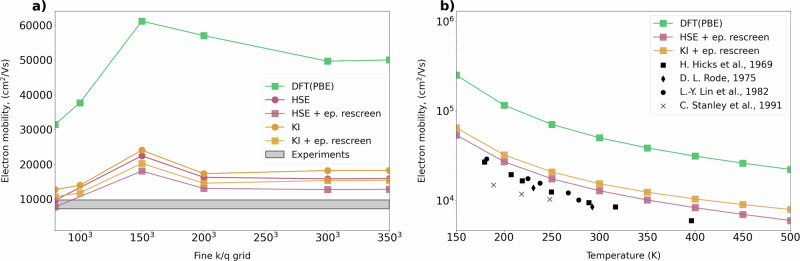
Mobility with beyond-DFT approaches. **a** Convergence of electron drift mobility of GaAs at room temperature with different functionals. The results with circles are obtained by only changing the band structure. In contrast, the results with squares also consider the effects of beyond-DFT functionals on electron-phonon coupling by introducing average rescreening of the electron-phonon matrix. The experimental results are in good agreement with HSE and KI functionals. **b** Temperature dependence of electron drift mobility of GaAs with different functionals. Black markers represent experimental results that lie very close to beyond-DFT values.

In the case of GaAs, we find that its electron mobility exhibits a significant change compared to that of DFT when calculated with beyond-DFT approaches. From Fig. 3, we see that the effect of advanced functionals is crucial to obtain good agreement with experimental results^76–82^. It is important to note that the long-range contribution to the electron-phonon coupling is currently treated at the DFT (PBE) level, as further methodological developments are required to extend this treatment beyond standard DFT. Additional tests indicate that an improved dielectric constant can modify the long-range electron-phonon interaction and further reduce the carrier mobility, bringing the predicted value into closer agreement with experiment. See the SI^52^ for more discussions on this point. Overall, the main reason of improved result is the strong underestimation of the electron effective mass of GaAs in DFT^83^. HSE and KI functionals better predict the electron effective mass, and this significantly improves the mobility. The secondary effect is the renormalization of electron-phonon couplings, which gives an additional contribution of around 20–30% with respect to DFT electron-phonon interactions. An important conclusion from the calculation of the mobility of GaAs is the significance of band curvature. Table 2 provides the effective mass on the band edges for Si and GaAs that are used in the calculations. Starting from Si, one can see that the HSE and *G*_0_*W*_0_ are doing well in describing the transversal component of the electron’s effective mass if compared to DFT or KI. However, they worsen the longitudinal values for electrons and light holes. The KI functional does not change significantly the effective masses for this system, and in particular does not modify the hole effective masses. This is due to the fact that the 4 *sp*^3^ Wannier functions spanning the occupied manifold, and representing the localized manifold of the Koopmans construction, happen to have the same KI correction, leading to a basically rigid shift of the valence manifold without any modification of the dispersion (**k**-dependence). This is not the case for the empty-state manifold, where changes in the band dispersion are observed. While the rigid shift of the bands is observed here for Si (and similar systems), we stress that it is not a general feature. A different choice of the Wannier manifold^84^ or the use of more advanced Koopmans flavors, such as KIPZ or pKIPZ, would introduce a non-trivial and **k**-dependent correction to the DFT bands for both occupied and empty states. We defer the reader to SI^52^ for more information.

**Table 2:** Effective masses for Si and GaAs with different methods (in units of the free electron mass, *m*_*e*_)

Material	Method	Electron ${m}_{| | }^{* }$	Electron ${m}_{\perp }^{* }$	Hole ${m}_{hh}^{* }$	Hole ${m}_{lh}^{* }$
Si	DFT	0.859	0.202	0.276	0.177
	HSE	1.099	0.189	0.276	0.185
	KI	0.820	0.208	0.276	0.177
	*G*_0_*W*_0_	0.905	0.198	0.284	0.188
	Experiment^67^	0.920	0.190	0.490	0.160
GaAs	DFT	0.027		0.316	0.027
	HSE	0.058		0.304	0.057
	KI	0.054		0.316	0.027
	Experiment^67^	0.067		0.510	0.080

Both HSE and KI significantly improve the isotropic electron effective mass of GaAs. HSE provides improved value for the light-hole effective mass, but for accurate transport calculation, it is necessary to include spin-orbit coupling. These results support the general conclusion that the description of electron mobility benefits from the usage of beyond-DFT functionals.

## Discussion

In this work, we introduced a novel framework to calculate electron-phonon couplings that works with any electronic-structure method, as long as it can provide electronic eigenvalues and eigenvectors of perturbed and unperturbed systems. The approach is general, allows integration with many modern DFT and beyond-DFT methods, and is elegant in bypassing the challenges of direct methods (orbital-dependent operators) through a novel projectability approach.

Using symmetry considerations, we devised an efficient algorithm to unfold wave functions to all required supercells, drastically decreasing the number of calculations needed to perform. The whole approach is implemented in a new dedicated electron-phonon code named ElePhAny that is interfaced with the Quantum ESPRESSO and the KOOPMANS packages, as well as YAMBO, and allows the evaluation of electron-phonon coupling for all functionals available in the mentioned codes. After that, an interface to the EPW code allows the calculation of any electron-phonon-related properties that require fine sampling of the Brillouin zone.

We used the proposed approach to study the electron-phonon coupling, effective masses, and mobilities in Si and GaAs. Using advanced electronic structure methods leads to a significantly improved description of electron mobilities in GaAs and results in a closer agreement with experimental results. The proposed approach paves the way for the calculation of electron-phonon-related properties with a realistic electron description.

## Methods

### Computational workflow

**Fig. 4:**
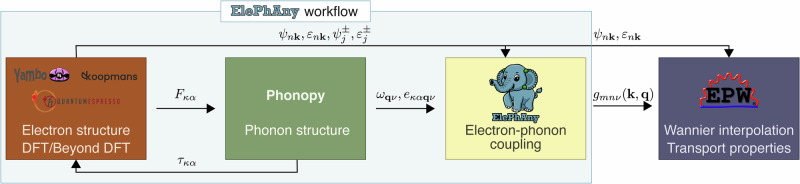
Schematic representation of the workflow. The ElePhAny code serves as the main driver for orchestrating the preceding calculations of electronic structure and phonon properties, as well as for calculating electron-phonon matrix elements using the projectability approach. As the initial step, the code calls the PHONOPY application programming interface to create displaced supercell configurations based on the input of the preferred electronic structure code (Quantum ESPRESSO, KOOPMANS, YAMBO). After running the chosen electronic structure code and providing a set of resulting wave functions (in binary or HDF5 format) and eigenvalues in XML format together with Phonopy output of phonon eigendisplacements and frequencies in YAML format, the ElePhAny code constructs an electron-phonon matrix and saves it in the binary EPW .epb format for further calculation of electron-phonon related properties.

Calculating transport properties using the proposed approach requires several steps. Starting with the atomic structure, one (i) performs self-consistent calculations for the pristine and perturbed systems, (ii) calculates phonon dispersion, (iii) computes electron-phonon matrix elements on a coarse grid, (iv) Wannierizes target manifold and interpolates all relevant quantities on a fine grid, and (v) solves the Boltzmann transport equation with the EPW code. Some of these tasks are achieved by using existing codes. In particular, we use Quantum ESPRESSO^40,41^, KOOPMANS^42^ and YAMBO^43^ for DFT, hybrid, Koopmans and *G*_0_*W*_0_ calculations, PHONOPY^37^ for the generation of supercells with displaced atoms and phonon dispersion, and EPW^44,45^ for Wannier interpolation and BTE solution. For the remaining tasks, we developed a flexible and modular JULIA code called ElePhAny that evaluates the electron-phonon matrix elements on a coarse grid with the projectability approach, and interfaces and manages the other software involved to obtain all the quantities required for the electron-phonon matrix elements calculations. The overall workflow is depicted in Fig. 4. Although we used the Quantum ESPRESSO package, we stress that the approach is general and can be applied to any first-principles software that provides eigenvalues and forces. The electron-phonon matrix elements in the KS basis are saved in a binary format readable by EPW, which is used for the subsequent Wannier interpolation and transport properties calculation. A PHONOPY interface is used to generate the inputs for the perturbed supercell calculations. Since phonon properties could also be computed using only inequivalent displacements, the ElePhAny code checks whether the proposed displacements would allow to obtain all perturbed configurations using Eq. (10). After having performed supercell calculations, the most time-consuming part of the evaluation of the electron-phonon matrix element via the projectability approach is the evaluation of Eq. (4) for each **k** and **q** points. Although we do not exploit it here, we note that this could be efficiently parallelized over **k** points. For more details on the gain in computational cost from the use of symmetries in FD calculations, see Fig. 1 of the SI^52^.

### Computational details

To demonstrate the approach, we performed electron-phonon and mobility calculations for Si and GaAs using PBE, HSE, and KI functionals. For both systems, we used supercells of sizes 2 × 2 × 2, 3 × 3 × 3, and 4 × 4 × 4 to demonstrate the convergence of rescreening of the electron-phonon matrix elements. In both calculations, the hybrid functional was chosen as HSE06^26,85^ with a fraction of the exact exchange set to the default of 0.25. For Si, we also performed *G*_0_*W*_0_ calculations in the pristine unit cell, as well as in 2 × 2 × 2 and 4 × 4 × 4 supercells with displaced atoms. The finite displacement step was chosen to be 0.001 Bohr to ensure convergence of the FD approach.

For Si, we used an optimized norm-conserving Vanderbilt pseudopotential^86,87^ from the DOJO library^88^, wavefunctions cutoff of 60 Ry. For Wannier interpolation of band structure and electron-phonon matrix elements, we used sp^3^ orbitals as initial projections for occupied bands and *s* + *d* for empty ones. To perform the disentanglement procedure, we calculated 20 bands. In the case of the Koopmans KI functional, the same initial projections were chosen as variational orbitals for primitive cell calculations. From the tests, we observed that even though the effective mass is significantly affected by the quality of interpolation, the electron-phonon coupling with the Koopmans functional was much less sensitive to that. For that reason, supercell calculations were performed with sp^3^ orbitals as initial projections for empty bands to reduce the computational cost to 4 empty orbitals. The screening *α* parameters were calculated with a linear response scheme^84,89^ on the 6 × 6 × 6 grid and fixed for the Hamiltonian calculation with finer grids. For the final mobility calculation, the coarse grid of 12^3^ ensures convergence of effective mass for electrons. Such a large coarse grid was required since the conduction band minima do not lie on a high symmetry point^67^. For the fine grids, values of 60^3^ and 100^3^ were chosen. The *G*_0_*W*_0_ calculation was performed using 100 states of the corresponding pristine unit cell (6400 states in the 4 × 4 × 4 supercell). For the dielectric function evaluation, we rely on the plasmon pole approximation^90^ and use G-vectors cutoff of 8 Ry for dielectric matrix.

For GaAs, a Schlipf-Gygi optimized norm-conserving Vanderbilt pseudopotential^91^ with wavefunctions cutoff 80 Ry was used. However, for the HSE functional, we had to use the cutoff of 100 Ry to overcome the issue of soft modes in phonon calculations with hybrid functional. For both occupied and empty bands, sp^3^ orbitals were used. Moreover, the wannierization was performed by mixing occupied and empty bands to improve the localization by increasing the number of degrees of freedom in the minimization procedure. KI functional was also built on top of the Wannier functions with sp^3^ orbitals as initial projections. The screening parameters were calculated on the 4 × 4 × 4 grid and then fixed for further Hamiltonian calculations. In this case, the orbitals are not mixed, since the Koopmans functional tends to favor block-by-block wannierization^92^. As the final coarse grid for obtaining eigenvalues of the Hamiltonian and rescreened electron-phonon coupling, the 8^3^ sampling and 60^3^–350^3^ fine grid were chosen.

## Supplementary information

Supplementary Information

## Data Availability

The data needed to reproduce the results in this work can be found in the Materials Cloud Archive^93^.

## Code availability

The source code is available on GitHub (https://github.com/Koulb/ElePhAny.jl).
