# Supplementary material for: Carrier mobilities and electron-phonon interactions beyond DFT
Source: NPJ Comput Mater. 2026 Mar 3;12(1):151. doi: 10.1038/s41524-026-02011-2 (PMC13076207; doi:10.1038/s41524-026-02011-2)
Supplement: Supplementary file 1 — Supplementary Information [file 41524_2026_2011_MOESM1_ESM.pdf]

Supplementary Information:
Carrier mobilities and electron-phonon interactions beyond DFT

Aleksandr Poliukhin,[1] Nicola Colonna,[2] Francesco Libbi,[3] Samuel Poncé,[4,5] Nicola Marzari,[1,2]
Aleksandr Poliukhin,[1] Nicola Colonna,[2] Francesco Libbi,[3] Samuel Poncé,[4,5] and Nicola Marzari[1,2]
*Theory and Simulation of Materials (THEOS), École polytechnique fédérale de Lausanne, 1015 Lausanne, Switzerland**
PSI Center for Scientific Computing, Theory and Data, 5232 Villigen PSI, Switzerland
John A. Paulson School of Engineering and Applied Sciences,
Harvard University, Cambridge, MA 02138, USA
European Theoretical Spectroscopy Facility, Institute of Condensed Matter and Nanosciences,
Université catholique de Louvain, Chemin des Étoiles 8, B-1348 Louvain-la-Neuve, Belgium
WEL Research Institute, avenue Pasteur 6, 1300 Wavre, Belgium
(Dated: January 15, 2026)

WANNIER INTERPOLATION OF THE ELECTRON-PHONON MATRIX ELEMENTS IN THE CASE OF POLAR MATERIALS

For the long-range contribution, we calculate the dielectric tensor and Born effective charges at the DFT level, since beyond-DFT methods would require a separate procedure at finite electric field [1]. Moreover, in all cases of the beyond-DFT methods, one should additionally consider the long-wavelength limit of the additional part of the effective potential (due to exact exchange (HSE), orbital-density dependent potential (KI), and self-energy (GW)). Considering, for now, that long-range contribution comes only from the PBE exchange-correlation potential, we show the maximum effect of the changes due to different treatment of the long-range contribution to the electron-phonon coupling. To that end, we performed the final calculation of the electron mobility of GaAs at the DFT level, using a dielectric constant calculated at the DFT level (15.1) and an experimental value [2] (10.86) demonstrated on Fig. S1. This lowers the mobility by an additional 15 %, showing that this is a smaller effect than the ones considered in the main text.

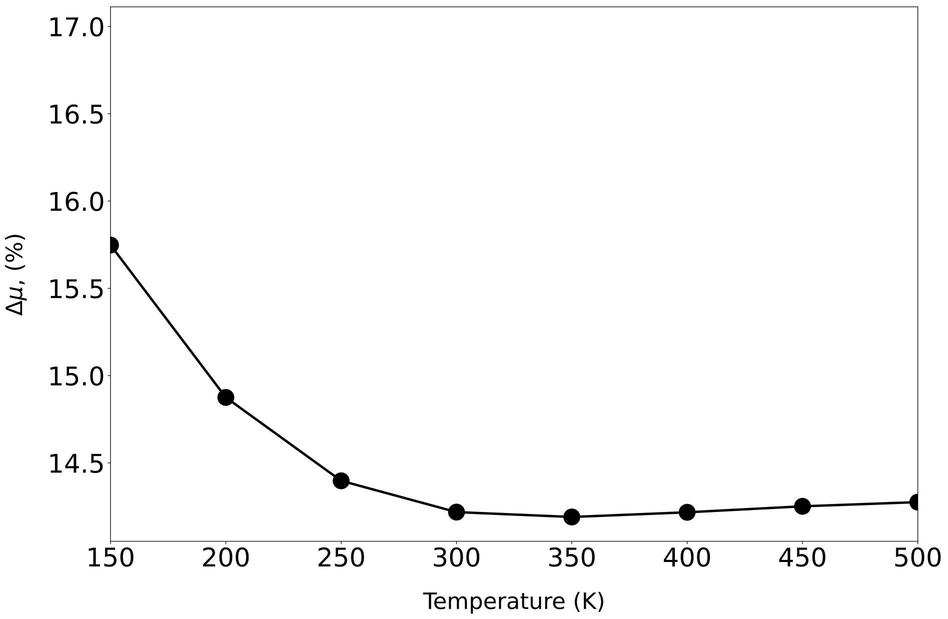

FIG. S1. Relative change in the electron mobility of GaAs when long-range part of the electron-phonon coupling is treated with DFT and experimental dielectric constant.

* aleksandr.poliukhin@epfl.ch

FINITE DIFFERENCE APPROACH WITH DERIVATIVES OF THE POTENTIAL

In a conventional finite difference approach, the derivatives that are required in electron-phonon matrix elements could be calculated as the finite difference of the effective potential. The caveat in such an approach is that the total potential often consists of local and non-local parts, where the latter is due to standard pseudopotential construction [3, 4]. Therefore, an individual treatment is required for different types of pseudopotentials. Recent works in this direction show that the expression for PAW pseudopotentials can be challenging to implement [5]. In the present work, we considered only norm-conserving pseudopotential, for which we derived the following contribution of the non-local part of the potential to the corresponding brakets [6]: Fig. S2 shows that the projectability and finite difference (FD) of the potentials approaches give a comparable accuracy compared to DFPT.

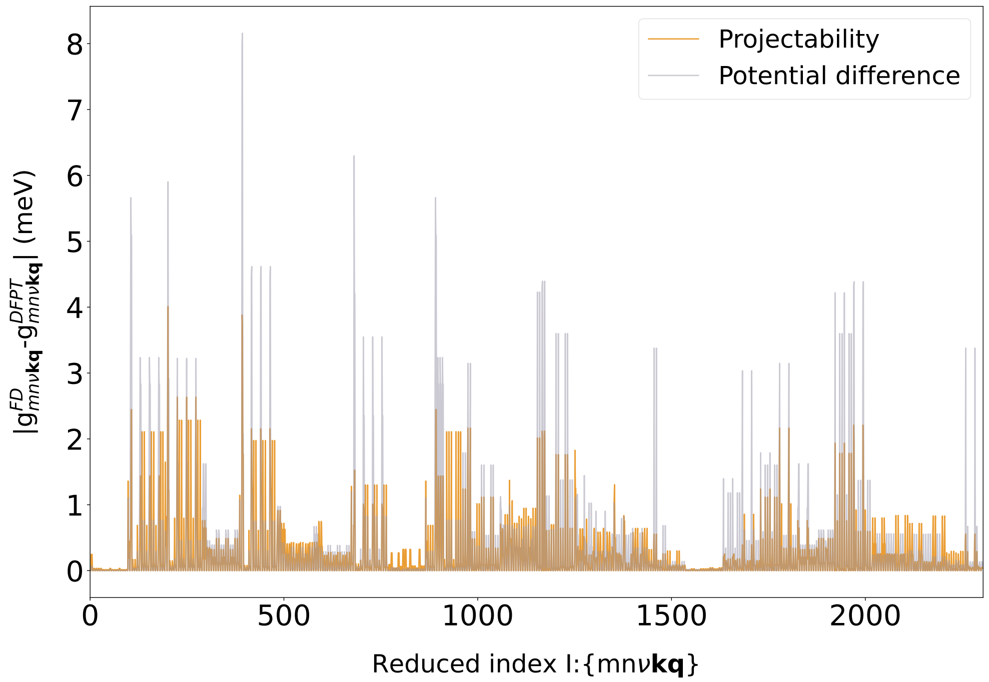

FIG. S2. Comparison of 2 finite-difference (FD) approaches: the projectability approach described in the paper and the conventional potential differences for a 2×2×2 supercell of Si with absolute displacement of 10^{-2} Bohr. The error of both methods lies in the same range, even though the projectability approach is more accurate for some values.

CONVERGENCE OF FINITE DIFFERENCE APPROACH

To ensure the reliability of our calculations, we performed various convergence tests. Fig. S3 illustrates the typical convergence behavior of electron-phonon matrix elements as a function of the finite difference step size. Here, as well as in all production run calculations, second order finite difference is used. The results show that convergence is achieved for step sizes smaller than $\delta\tau < 10^{-2}$ Bohr. Beyond this point, the convergence reaches a plateau and does not improve any further. This plateau for FD methods is well known and stems from numerical accuracies [7, 8]. This can introduce minor inconsistencies between FD and DFTP that prevent the exact numerical agreement of the electron-phonon matrix. However, the error remains consistent with what is typically observed when comparing quantities such as the electron-phonon matrix element across different numerical setups and codes [9, 10]. The FD projectability formula proposed in this work can still show a convergence trend approaching numerical precision. In the case of the non-degenerate band at $\mathbf{q} = \Gamma$, the FD projectability approach reduces to the eigenvalue differences divided by the absolute displacement. By examining the specific case where DPFT predicts this braket to go to zero, we can analyze the convergence of the FD approach. This is shown in Fig. S4 where we examine the convergence of the eigenvalue difference for a selected silicon band ($m = n = 1$, $\mathbf{k} = \Gamma$). We see that the eigenvalue difference goes to zero faster than the displacement step as the latter goes to zero. Additionally, the effect of using different discretization schemes is also presented in Fig. S4. When first-order FD is used, we need to go to minimal displacements to converge whereas second-order FD delivers numerical accuracy for all the range of FD considered. Another critical point is that, in such calculations, second-order finite differences should be used, because they have a smaller requirement for the convergence of the total energy (conv_thr). Since most modern DFT codes check convergence of the total energy, not eigenvalues, they are, in principle, not guaranteed to converge to the same accuracy. In the present case, convergence to numerical precision of 10^{-16} with first-order finite difference could be only achieved when conv_thr is set to 10^{-30}, which is highly computationally demanding. Second-order finite differences give the same result with less strict conv_thr=10^{-13}, stressing once again the importance of using it not only because of the reduced error with respect to the discretization step, but also because it significantly reduces the requirements for accuracy of self-consistency. We remark that we did not use crystal symmetries for these calculations since QUANTUM ESPRESSO would detect them to be the same when the displacements are smaller than 10^{-5} Bohr.

To further assess the accuracy of our FD approach compared to DFPT, it is also important to check the consistency between electron-phonon matrix elements produced by EPW after performing the Wannier interpolation when using the conventional DFPT or the FD interface proposed in this work. This is illustrated in Fig. S5. Most of the electron-phonon matrix elements agree with an error of less than 0.01 meV, which allows to predict the same DFT mobility when using both schemes.

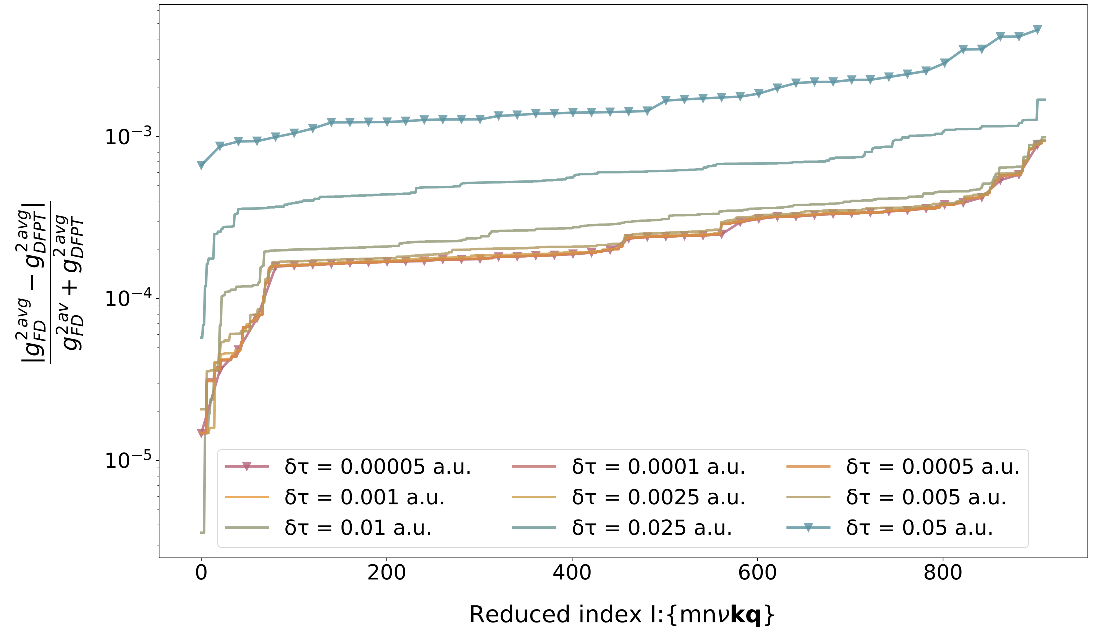

FIG. S3. Relative convergence of FD approach with respect to finite difference step for the 2×2×2 **q**-grid of silicon. It is seen that the finite step below or equal to 10^{-3} Bohr allows the convergence of most of the electron-phonon matrix elements to the relative error of 10^{-3}–10^{-4}.

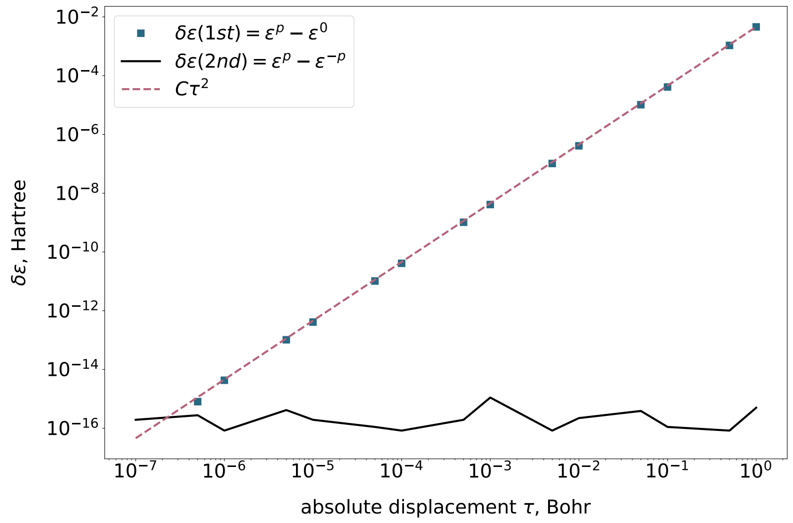

FIG. S4. Difference between perturbed eigenvalues computed with different finite difference schema for the first energy level of silicon at $\mathbf{k}$=Γ. The electron-phonon matrix element should be zero for this band so it represents the convergence of the finite difference approach in this specific case.

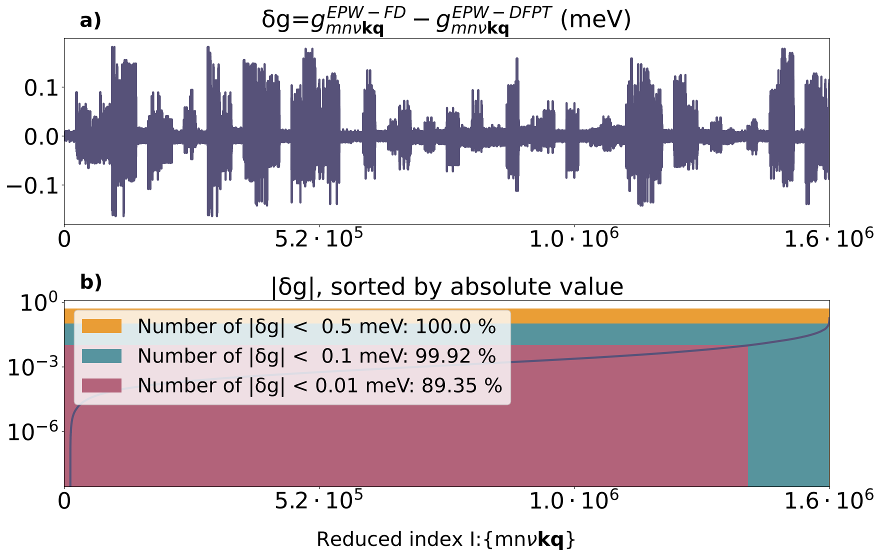

FIG. S5. The error of matrix elements produced by EPW, using FD and DFPT as an initial method for 4×4×4 **q**-grid of GaAs. We report both **a)** absolute error and **b)** modulus of error sorted by absolute values. Most of the electron-phonon matrix elements differ by less than 0.01 meV, which allows for the correct reproduction of results for mobilities on the DFT level.

ELECTRON-PHONON MATRIX ELEMENTS WITH BEYOND-DFT METHODS

The phonon band structure of Si with different beyond-DFT methods are shown in Fig. S6. The direct calculation of the electron-phonon matrix element for the first 4 unoccupied bands for $\mathbf{q}$=$\boldsymbol{\Gamma}$ and $\mathbf{k}$ along the high symmetry line $\boldsymbol{\Gamma L}$ is shown in Fig. S7. The limited number of points is due to the 4×4×4 supercell used in these direction calculations. It is worth mentioning that the HSE and KI functionals introduce a smooth enhancement of the coupling in this case, in contrast to G_0W_0, which increases and decreases the coupling depending on the band index. Interpolated on the $\boldsymbol{X}-\boldsymbol{\Gamma}-\boldsymbol{L}$ path using EPW, the electron-phonon coupling of GaAs is presented in Fig. S8. The correction of beyond-DFT methods also behaves like a smooth function.

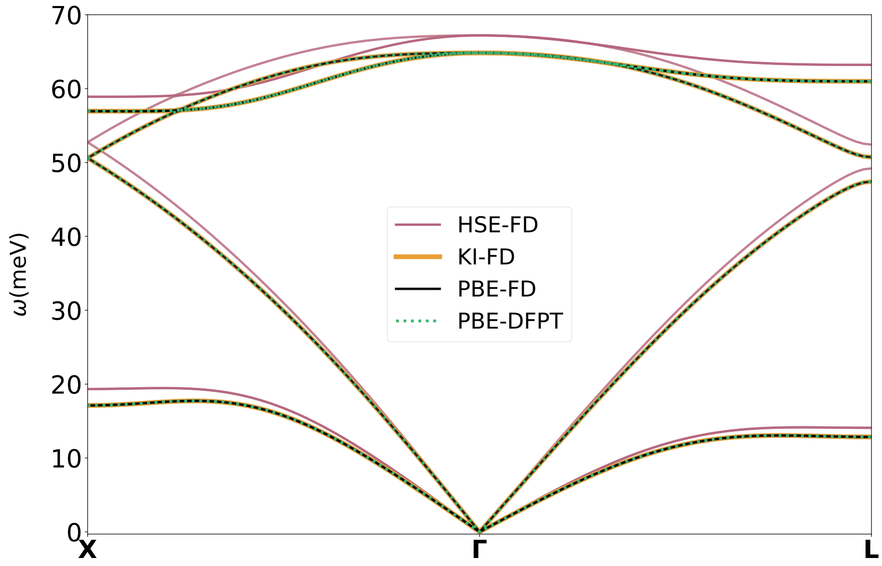

FIG. S6. Phonon band structure of Si with different functionals. The interpolation is performed from a 4×4×4 supercell.

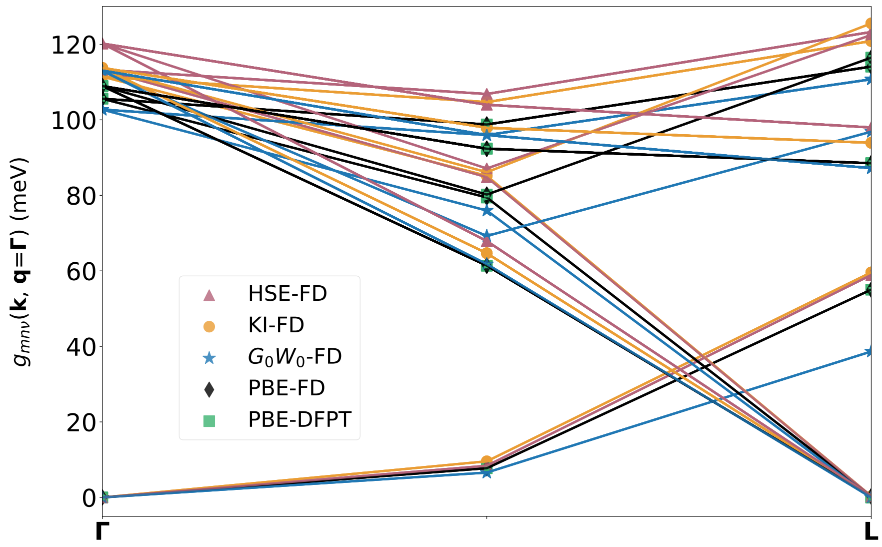

FIG. S7. Electron-phonon matrix elements of Si with different functionals for the first 4 occupied bands for $\mathbf{q}$=$\boldsymbol{\Gamma}$ and $\mathbf{k}$ along the high symmetry lines $\boldsymbol{\Gamma}-\boldsymbol{L}$. HSE and KI functionals yield an uniform increase in the values of the electron-phonon matrix elements compared to DFT.

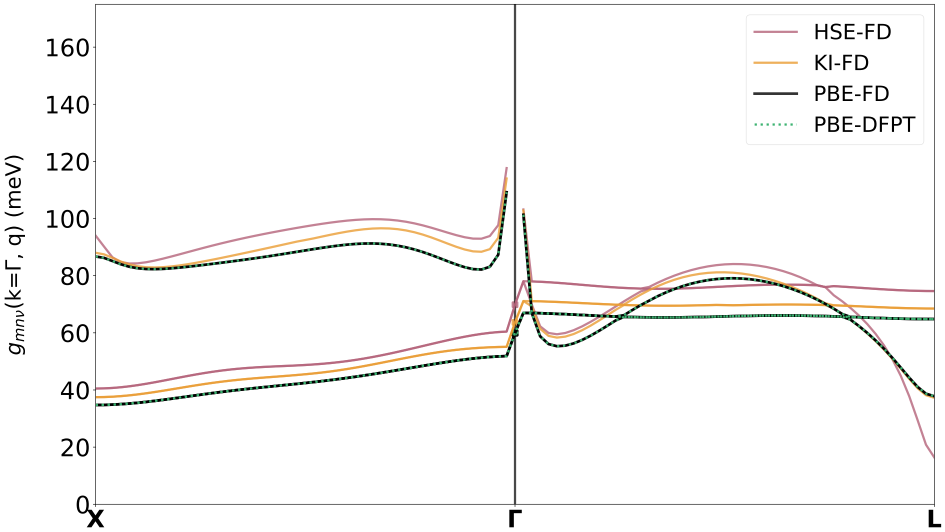

FIG. S8. Electron-phonon matrix elements of GaAs for $n = m = 4$ optical branches with different beyond DFT functionals. Interpolation starting from a $4{\times}4{\times}4$ supercell.

AVERAGE RESCREENING OF THE ELECTRON-PHONON MATRIX ELEMENTS

The main idea behind rescreening lies in the observations that were made on the rate of convergence of mobilities with different functions. Numerical tests show that the ratio between DFT and beyond-DFT mobility converges more quickly than the mobility itself. Moreover, the ratio between the DFT and beyond-DFT electron-phonon matrix elements exhibits the same trend. Since mobility is inversely proportional to the square of the electron-phonon matrix elements, it is reasonable to assume that the ratio between these quantities is in agreement. This is illustrated in the table S1. The rescreening factor and the mobility reduction factor agree reasonably well and converge rapidly with supercell size.

To elaborate on why this is happening, one could look at the enhancement of the electron-phonon matrix elements due to the beyond-DFT function depicted in Fig. S9. We see that the rescreening of the electron-phonon matrix elements occurs within a narrow window, which allows for the introduction of a single rescreening factor in the final calculation of the mobility. Since the G_0W_0 correction requires a sum over a large number of empty states, obtaining converging trends with different supercells is more challenging. This is why, for the G_0W_0 calculation, we determined rescreening based on the largest supercell, which still remains affordable for the calculation. The same trend of rescreening convergence is observed when we look at the mobility of Si, as shown in Figs. S10 and S11. for KI and HSE functionals, respectively. For the occupied bands, the KI correction is a constant shift, and for the comparison between DFT and HSE, we fixed the eigenvalues at the HSE level to neglect the change in the effective mass. These two figures represent the contribution of only beyond-DFT electron-phonon coupling to mobility. As expected, the increased values of the electron-phonon coupling reduce mobility since the latter is inversely proportional to the scattering rates. The reduction itself remains almost constant, justifying the use of a rescreening factor. Since the rescreening factor determined from averaging of electron-phonon couplings could be obtained directly from a coarse grid, we use it instead of the mobility reduction factor in all of the calculations since the latter requires reasonable Wannier interpolation, which is hard to achieve in the case of small supercells. Using this rescreening factor allows us to bypass the calculation of electron-phonon matrix elements on prohibitively large supercells, which would otherwise be required to match the $\mathbf{k}$-grid necessary to converge the effective masses. Importantly, since in the present work we only consider the transport property, an alternative strategy could be rescreening only in the vicinity of the band edges, which account for the essential region for mobility calculation. We found that this procedure yields results similar to those obtained with the original method. The restricted rescreening area only includes the bands that correspond to valence band maximum (VBM) and conduction band minimum (CBM), $\mathbf{k}$ points that correspond to the positions of band edges, and $\mathbf{q}$ that satisfy the condition $|\mathbf{q} - \mathbf{q}^{VBM/CBM}| \leq 0.15$. Comparing with table S1, we have that for HSE, averaging over all bands gives 1.074, while averaging near the valence band maximum (VBM) gives 1.0595 and near the conduction band minimum (CBM) 1.085. Similarly, for KI, we have averaging over all bands gives 1.046, while CBM and VBM give 1.023 and 1.049, respectively. While this is not a general statement and should be checked for the system at hand, the fact that the rescreening behaves similarly across different bands allows, in the future, to study properties that depend on the values of the electron-phonon coupling throughout the Brillouin zone, like e.g., optical absorption, band gap renormalization. To illustrate the effect of the average rescreening, we also compared the electron-phonon coupling of Si computed directly with beyond-DFT methods and the DFPT result rescreened by the factor obtained from smaller supercell calculations. As shown in the Fig.S12, the rescreened electron-phonon coupling closely matches the direct calculations, thus justifying the procedure for the production run.

TABLE S1. Rescreening of electron-phonon matrix elements and a corresponding reduction of the hole mobility for Si using KI or HSE with respect to DFT(PBE) functional. Since the effective mass stays here on the DFT level, the only contribution is a change in electron-phonon coupling.

Grid	Mobility reduction (DFT/KI)	$\left\|\left\langle \frac{g^{\mathrm{KI}}_{mn\nu}(\mathbf{k},\mathbf{q})}{g^{\mathrm{DFT}}_{mn\nu}(\mathbf{k},\mathbf{q})} \right\rangle\right\|^2$	Mobility reduction (DFT/HSE)	$\left\|\left\langle \frac{g^{\mathrm{HSE}}_{mn\nu}(\mathbf{k},\mathbf{q})}{g^{\mathrm{DFT}}_{mn\nu}(\mathbf{k},\mathbf{q})} \right\rangle\right\|^2$
2^3	1.046	1.031	1.065	1.092
3^3	1.058	1.040	1.043	1.166
4^3	1.046	1.038	1.074	1.189

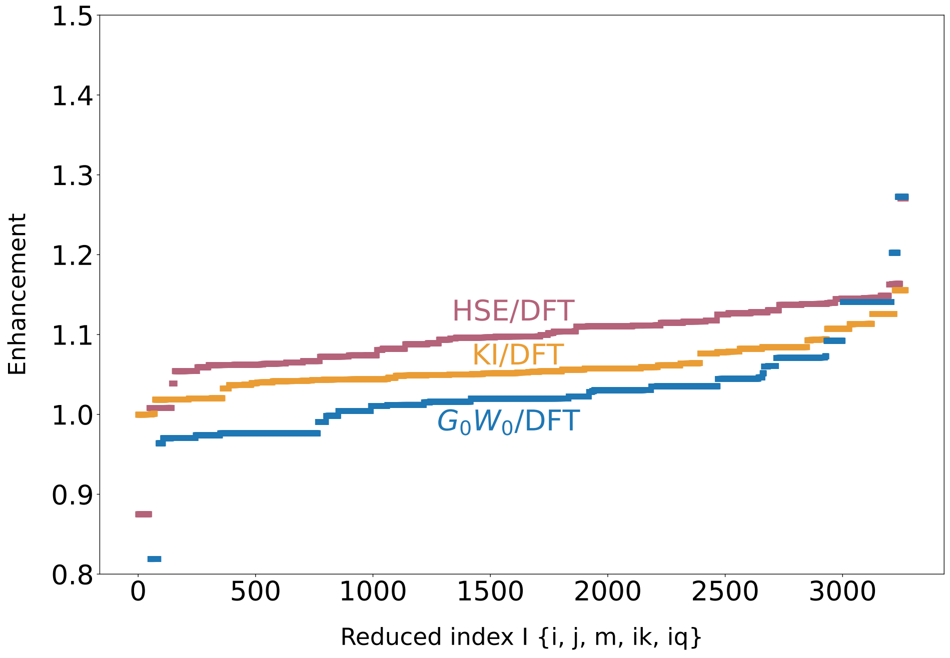

FIG. S9. Enhancement of the HSE, KI and G_0W_0 electron-phonon matrix elements of Si for the 2×2×2 supercell. The correction of beyond-DFT functionals with respect to DFT is reasonably well approximated by a constant factor for simple systems.

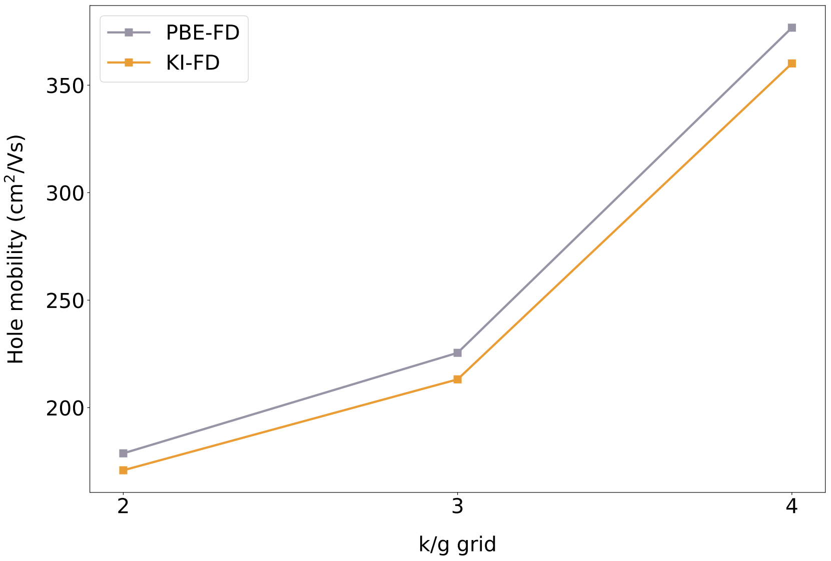

FIG. S10. Convergence of hole mobility of Si with respect to the coarse $\mathbf{k}/\mathbf{q}$ grid, which is equivalent to the supercell size of the FD approach. Since the effective mass for the holes stays on the DFT level, the only contribution is due to the renormalization of electron-phonon matrix elements.

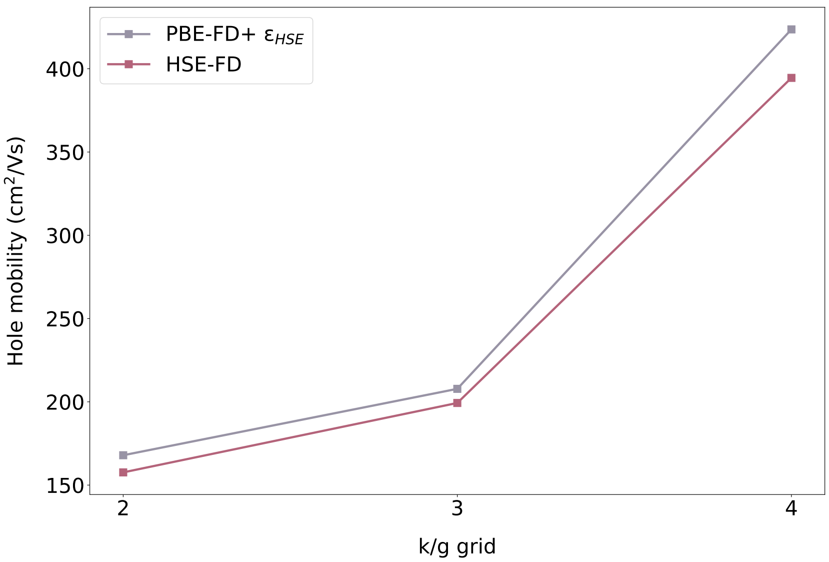

FIG. S11. Convergence of hole mobility of Si with respect to the coarse $\mathbf{k}/\mathbf{q}$ for HSE and PBE functionals. In the PBE-FD calculation we replace the PBE eigenvalues with the HSE one so that the effective mass in the two calculations is the same and the difference between the two calculations is only due to the renormalization of the el-ph matrix elements.

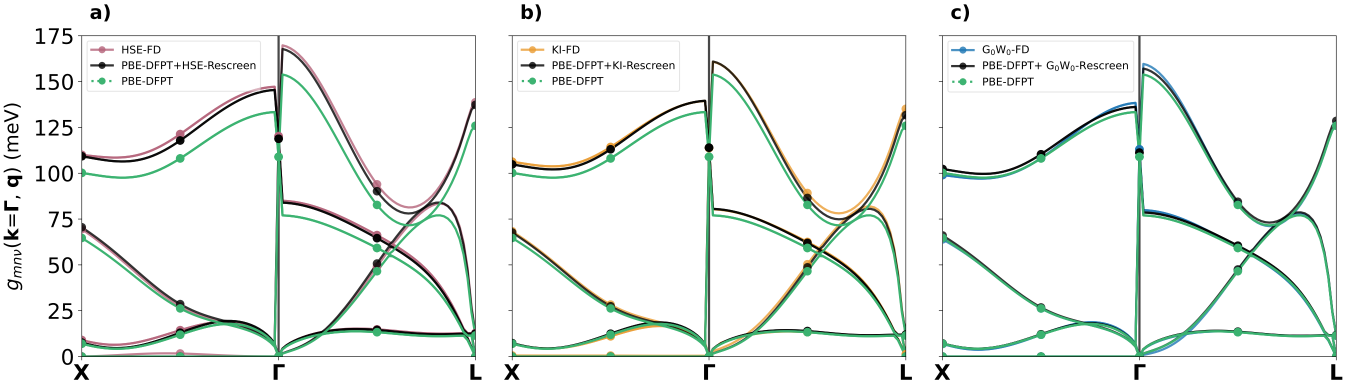

FIG. S12. Difference between the electron-phonon coupling of Si calculated by DFPT, by beyond-DFT methods: (**a**) HSE, **b**) KI, **c**) G_0W_0), and by applying average rescreening to the DFPT result. The couplings were calculated on the 4×4×4 grid, whereas the rescreening factors were determined from the smaller supercells (2×2×2), thereby justifying the procedure.

TEMPREATURE DEPENDENCE OF MOBILITY BEYOND DFT FOR SI

Fig. S13 shows the temperature-dependent hole and electron mobility of Si calculated with different methods: DFT(PBE), HSE, Koopmans(KI), and G_0W_0 compared against experimental data [11–15]. Consistent with the room-temperature results, beyond-DFT functionals tend to decrease mobility due to enhanced electron–phonon coupling and a more accurate description of the effective mass. For most experimental points, beyond DFT functionals, we obtain better agreement, except for a few points at low temperature for hole mobility. Bigger discrepancies in this case could be attributed to the absence of spin-orbit coupling in the calculation, which is more prominent for hole mobility in Si [16].

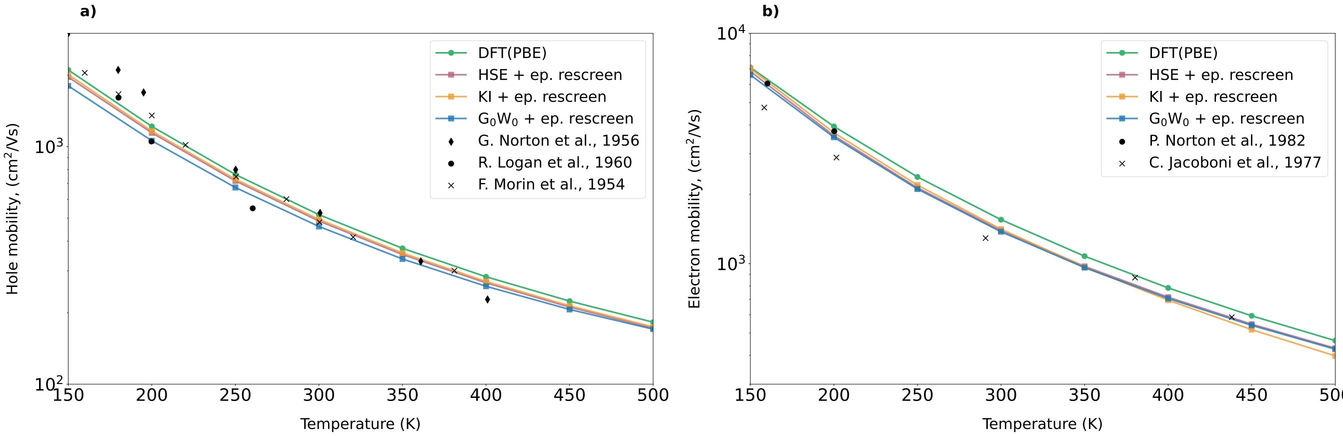

FIG. S13. Temperature dependence of **a)** electron and **b)** hole mobility of Si with beyond DFT methods. Black markers represent experimental results.

THE EFFECTIVE MASS WITH KI FOR DIFFERENT VARIATIONAL ORBITALS.

We provide here additional details on the structure of the KI Hamiltonian and on how this affects the dispersion (**k**-dependence) of the KI electronic band structure. Assuming the Wannier functions of the KI Koopmans correction comes from a block-by-block Wannierization producing subsets of identical Wannier functions (this is the case in Si where the Wannier functions are 4 sp^3-like orbitals), the corrective KI Hamiltonian in this basis amounts to a block-diagonal matrix with scalar blocks, meaning that the original DFT states will also be the ones that diagonalize the resulting total KI Hamiltonian and that the final KI eigenvalue will be rigidly shifted by the same amount inside each sub-blocks. This is exemplified for the case of GaAs in Fig. S14. In the general case where orbitals (5 semicore d-state and 4 s and p covalence states) are allowed to mix, the simplified correction described above does not apply, and one gets a correction to the band dispersion that is more complex than a constant shift. On the other hand, if Wannierization is performed block-by-block we arrive at a constant shift of the band structure. This illustrate the orbital-density-dependence of the Koopmans correction and poses the natural question of which set of Wannier functions we need to choose. To answer this question, we recalled the alternative definition of KI as a limit of KIPZ functional with vanishing PZ term. Without this definition, any choice of the Wannier function would deliver the same total energy since the KI energy functional for insulating systems is invariant with respect to unitary rotation and would be equally good choice from an energetic point of view (although producing different band structure as discussed above and in Fig. S14). Turning on even an infinitesimally small PZ term breaks the energy invariance and allow to distinguish between different sets of Wannier functions. Figure S15 shows the total energy of the pKIPZ functional of Si and GaAs when different sets of Wannier functions are used as proxy for the minimizing orbitals. Using sp^3-like Wannier function as approximated minimizing orbitals produces a lower total KIPZ energy that using a set of 1 s-like and 3 p-like Wannier functions, and justify the use of the former. As a final remark, we state that in the general case of a sub-block consisting of orbitals of different characters, the Koopmans correction to the eigenvalues becomes **k**-dependent.

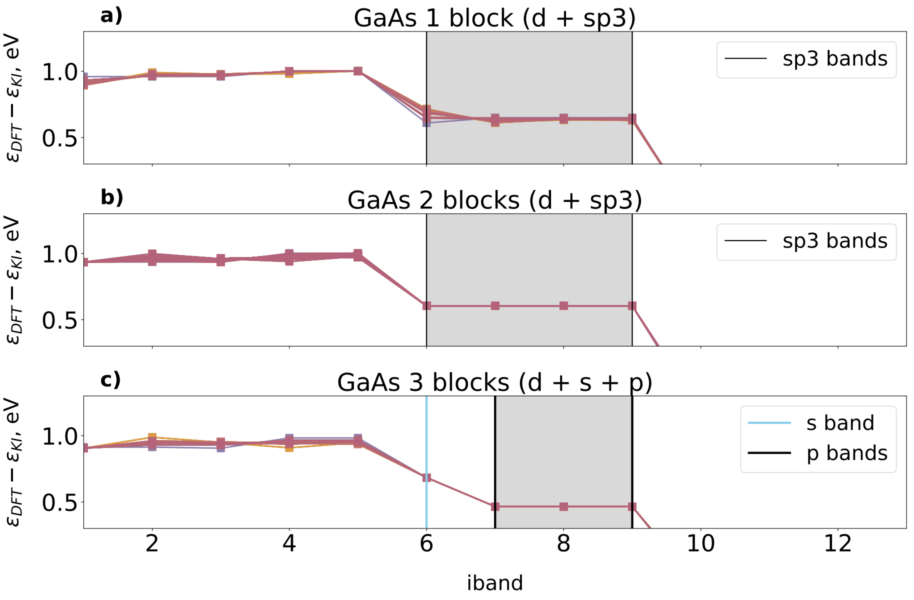

FIG. S14. The difference between DFT and Koopmans eigenvalues (shifted so that the VBM matches) for several GaAs states. Different colors represent different **k**-points. The choice of Wannier functions and the way orbitals are grouped might affect the curvature of the final KI band structure. **a** Wannierization is done in 1 block, allowing d and sp^3 orbitals to mix. **b** Wannierization is done in 2 blocks, separating d and sp^3 orbitals. **c** Wannierization is done in 3 blocks, separating d, s, and p^3 orbitals In the calculation where the Wannierization is performed block-by-block and all the orbitals inside the block have the same character (e.g., sp^3 or p^3) the correction represents just a constant shift. Note that bands coming from d-like Wannier functions will always display a modified dispersion if compared to the DFT one, as in cubic symmetry d states always splits into to set of t_{2g} and e_g states thus having different KI corrections.

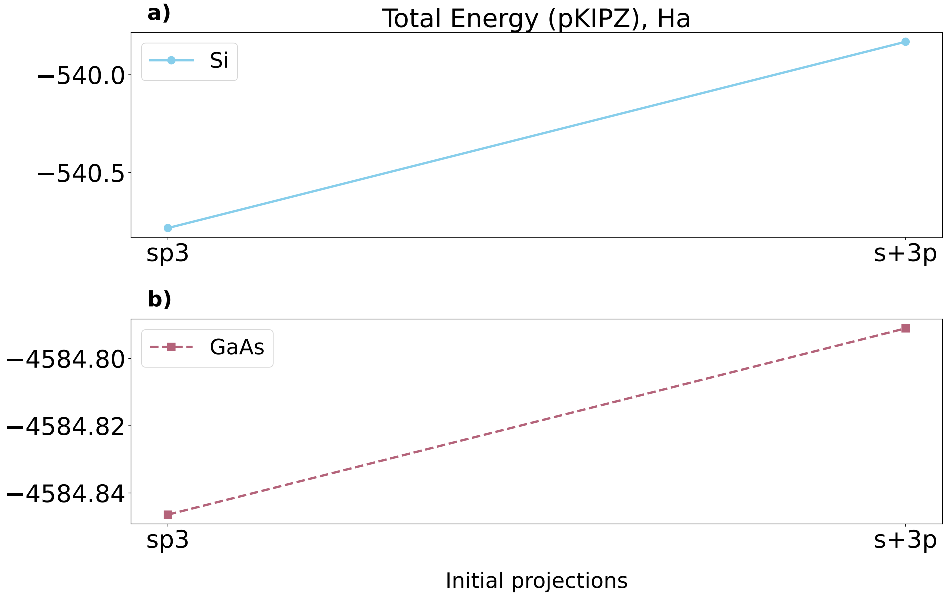

FIG. S15. The total energy of pKIPZ functional as a function of initial projection of occupied bands of **a)** Si and **b)** GaAs. In this case, functional favors to have Wannier functions of the same kind (sp3), delivering minimal energy.

COMPUTATIONAL COST

In this section, we provide quantitative estimates of the computational cost of the workflow described in the main text. All calculations were performed on the Eiger cluster at the Swiss National Supercomputing Centre (CSCS). The compute nodes used in this work have two sockets, each equipped with one AMD EPYC 7742 64-core processor (128 physical cores per node).

For the Si 4×4×4 supercell, the total wall-clock time for the full finite-difference workflow is presented in Fig. S16. The time spans a broad range, from approximately 2 to 2000 node-hours, depending on the specific electronic-structure setup (PBE, hybrid, G_0W_0, and Koopmans functionals), and the extent to which symmetries are exploited in the finite-difference calculations. In practice, the main limiting factor is the need to converge finite-difference supercell calculations with a beyond-DFT method. Importantly, we present estimates for both the FD and frozen-phonon approaches, where in the latter, for every phonon mode at every $\mathbf{q}$ point, an independent supercell calculation is required, significantly increasing the total computational cost. One caveat of using symmetry with FD is that, in the current implementation, direct calculation of 12 supercells of Si at the PBE level is cheaper than rotation of wave functions at different displacements, as discussed above, because the algorithm is not optimally parallelized. However, all methods beyond DFT significantly benefit from symmetry procedures, thereby reducing computational cost.

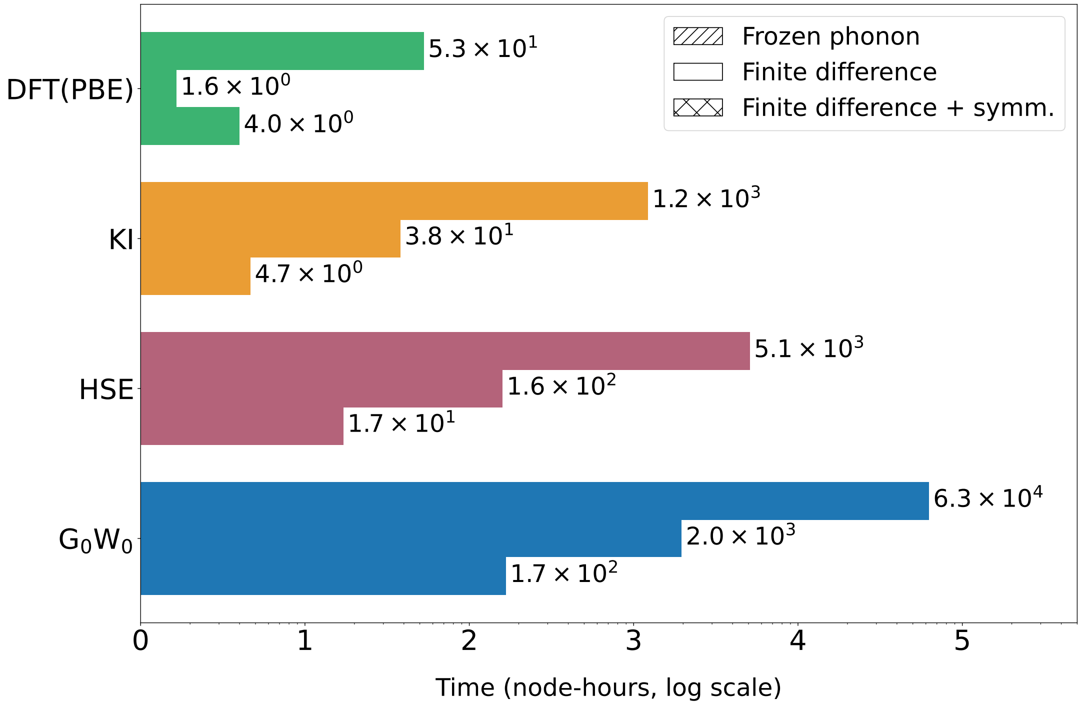

FIG. S16. Computational cost of electron–phonon coupling calculations for a 4 × 4 × 4 supercell of Si with beyond-DFT methods, using different symmetry and workflow options. For comparison, the final mobility calculation with rescreened DFPT electron–phonon couplings using EPW takes approximately 11 node-hours on the same architecture.

[1] L. Bastonero and N. Marzari, npj Computational Materials **10**, 55 (2024).
[2] W. J. Moore and R. T. Holm, J. Appl. Phys. **80**, 6939 (1996).
[3] L. Kleinman and D. M. Bylander, Phys. Rev. Lett. **48**, 1425 (1982).
[4] M. Van Setten, M. Giantomassi, E. Bousquet, M. Verstraete, D. Hamann, X. Gonze, and G.-M. Rignanese, Computer Physics Communications **226**, 39 (2018).
[5] L. Chaput, A. Togo, and I. Tanaka, Physical Review B **100**, 174304 (2019).
[6] F. Libbi, *Novel materials and algorithms for quantum technologies*, Ph.D. thesis, EPFL, Lausanne (2022).
[7] A. Togo and I. Tanaka, Scripta Materialia **108**, 1 (2015).
[8] J. Laflamme Janssen, Y. Gillet, S. Poncé, A. Martin, M. Torrent, and X. Gonze, Physical Review B **93**, 10.1103/physrevb.93.205147 (2016).

[9] S. Poncé, G. Antonius, P. Boulanger, E. Cannuccia, A. Marini, M. Côté, and X. Gonze, Computational Materials Science **83**, 341 (2014).
[10] S. Poncé, J.-M. Lihm, and C.-H. Park, npj Computational Materials **11**, 117 (2025).
[11] C. Jacoboni, C. Canali, G. Ottaviani, and A. Alberigi Quaranta, Solid-State Electronics **20**, 77 (1977).
[12] P. Norton, T. Braggins, and H. Levinstein, Phys. Rev. B **8**, 5632 (1973).
[13] F. J. Morin and J. P. Maita, Phys. Rev. **96**, 28 (1954).
[14] R. A. Logan and A. J. Peters, Journal of Applied Physics **31**, 122 (1960).
[15] G. W. Ludwig and R. L. Watters, Phys. Rev. **101**, 1699 (1956).
[16] S. Poncé, E. R. Margine, and F. Giustino, Physical Review B **97**, 10.1103/physrevb.97.121201 (2018), publisher: American Physical Society (APS).
